# Transsacadic Information and Corollary Discharge in Local Field Potentials of Macaque V1

**DOI:** 10.3389/fnint.2018.00063

**Published:** 2019-01-14

**Authors:** Michael A. Paradiso, Seth Akers-Campbell, Octavio Ruiz, James E. Niemeyer, Stuart Geman, Jackson Loper

**Affiliations:** ^1^Department of Neuroscience, Robert J. and Nancy D. Carney Institute for Brain Science, Brown University, Providence, RI, United States; ^2^Department of Applied Mathematics, Robert J. and Nancy D. Carney Institute for Brain Science, Brown University, Providence, RI, United States

**Keywords:** saccade, local field potential, LFP, support vector machine, corollary discharge, efference copy, V1, visual cortex

## Abstract

Approximately three times per second, human visual perception is interrupted by a saccadic eye movement. In addition to taking the eyes to a new location, several lines of evidence suggest that the saccades play multiple roles in visual perception. Indeed, it may be crucial that visual processing is informed about movements of the eyes in order to analyze visual input distinctly and efficiently on each fixation and preserve stable visual perception of the world across saccades. A variety of studies has demonstrated that activity in multiple brain areas is modulated by saccades. The hypothesis tested here is that these signals carry significant information that could be used in visual processing. To test this hypothesis, local field potentials (LFPs) were simultaneously recorded from multiple electrodes in macaque primary visual cortex (V1); support vector machines (SVMs) were used to classify the peri-saccadic LFPs. We find that LFPs in area V1 carry information that can be used to distinguish neural activity associated with fixations from saccades, precisely estimate the onset time of fixations, and reliably infer the directions of saccades. This information may be used by the brain in processes including visual stability, saccadic suppression, receptive field (RF) remapping, fixation amplification, and trans-saccadic visual perception.

## Introduction

Human visual perception takes place primarily during eye fixations that are separated by rapid saccadic eye movements. It is conceivable that perception and the mechanisms that control saccades are entirely independent processes, the saccades simply moving the eyes to objects of interest. However, multiple lines of evidence suggest that saccades influence visual perception. For example, just before saccades begin, visual space is compressed (Ross et al., 1997) and the spatial relationships between objects are altered (Cai et al., 1997). Saccadic suppression and our sense of perceptual stability across saccades may also rely on interactions between saccades and perception (Helmholtz, 1866/1911; Matin, 1974; Ross et al., 2001; Galletti and Fattori, 2003). Finally, there is considerable evidence that the brain’s guidance of visual attention piggybacks on the system used to move the eyes (Hoffman and Subramaniam, 1995; Deubel and Schneider, 1996; Mazer, 2011).

From a neural perspective, a foundation for understanding how perception and saccades interact should be based on establishing two key points. First, it must be shown that signals related to eye movements are present in brain areas involved in perception and, second, there must be information in the saccade-related signals sufficient to account for any perceptual effects. Numerous physiological studies in animals and humans have established the first point by showing that saccades are accompanied by changes in brain activity. Early EEG research showed that saccades are associated with changes in the activity of sensory cortex (i.e., lambda waves, Evans, 1953). More recent experiments have shown saccade-related neural activity in occipital BOLD signals, local field potentials (LFPs), and spiking activity (Bodis-Wollner et al., 2002; Purpura et al., 2003; Sylvester et al., 2005; Rajkai et al., 2008). Saccade-related changes in brain activity have been found in numerous visual areas including the LGN (Jeannerod and Sakai, 1970; Brooks and Gershon, 1971; Lee and Malpeli, 1998; Ramcharan et al., 2001; Reppas et al., 2002), occipital lobe (Wurtz, 1969; Tolias et al., 2001; Sylvester et al., 2005), temporal lobe (Sobotka et al., 1997), and parietal lobe (Pesaran et al., 2002; Kutz et al., 2003). Saccades have even been found to alter the functional connectivity between cortical areas (Sobotka et al., 2002).

The aim of the present study was to examine the second key point above, i.e., what information about saccades and fixations is present in peri-saccadic signals of early visual cortex? Of particular interest was information about the metrics of saccades (direction) and the timing of saccades and fixations. This information may be used by the brain in processes including visual stability (Helmholtz, 1866/1911), saccadic suppression (Matin, 1974; Ross et al., 2001), receptive field (RF) remapping (Duhamel et al., 1992), and trans-saccadic visual perception (Ross and Ma-Wyatt, 2004; Rajkai et al., 2008; De Pisapia et al., 2010; Ito et al., 2011; Paradiso et al., 2012). The presence in visual cortex of information about saccade metrics, such as direction, may be particularly important for visual stability which appears to require knowledge of saccadic eye movements (Wurtz, 2008). V1 signals conveying information about fixation onset timing may be critical for efficient visual processing. For example, there is evidence that visual processing at the start of new fixations may be enhanced by phase resetting–a “fixation amplifier”(Rajkai et al., 2008), or phase-locking of spikes to LFPs (Maldonado et al., 2008; Ito et al., 2011). Moreover, a striking degree of visual processing and perception appears to be based on a rapid feedforward sweep of information through the visual system. Evidence from experimental and theoretical studies suggests that initial visual recognition is based on the first one or very few spikes that neurons fire in each visual area at the start of a new fixation (Keysers et al., 2001; VanRullen and Thorpe, 2002). Thus, it may be critical that visual cortex “knows” precisely when a new fixation.

The present study explores signals in macaque primary visual cortex (V1) as this area plays critical roles in visual processing and perception. To focus on eye-movement related signals, and minimize confounds with visually-driven responses, visual stimulation was minimal. As the neurons did not generally fire action potentials in this situation, the analysis is based on recordings of LFPs. LFPs are also of interest because, unlike single-unit and multi-unit spiking, they are significantly correlated with BOLD signals in fMRI (Logothetis et al., 2001; Goense and Logothetis, 2008). Recordings were made with multi-electrode arrays so that simultaneous recordings at different cortical locations could be compared. To address the aims in an agnostic data-driven way, we used a simple linear support vector machine (SVM) approach to classify epochs of LFP activity. We find that LFPs in area V1 carry information that can be used to distinguish neural activity associated with fixations from saccades, precisely estimate the onset time of fixations (i.e., ends of saccades), and reliably infer the directions of saccades.

## Materials and Methods

### Experimental Subjects and Preparation

Two male rhesus macaques were used in these experiments (Monkey F weighed 10.4 kg, Monkey S weighed 9.9 kg). This study was carried out in accordance with the recommendations of the United States National Institutes of Health. The protocol was approved by the Brown University Institutional Animal Care and Use Committee. Niemeyer and Paradiso (2017) describe the methodology in detail. In separate aseptic surgical procedures, each animal was implanted with a custom titanium headpost and a 96-channel “Utah” array (Blackrock Microsystems). The array of 1 mm electrodes was placed in area V1 of the right hemisphere.

### Recording Procedures

Animals sat in a primate chair (Crist Instrument Co., Hagerstown, MD, USA) in a dimly-lit room. Their eyes were 63 cm from a CRT display (Iiyama Viewmaster HM204DT) that had a resolution of 1280 × 1024 and a refresh rate of 120 Hz. The visual display subtended 33 deg wide by 26 deg high and had a uniform luminance of 55 cd/m^2^. The only visual objects on this gray background were red fixation points with 0.25 deg radius; these points were never in the neurons’ classical RFs. To avoid visual stimulation from the far periphery, early experiments were conducted with a large foam core panel (78 deg wide by 65 deg high) surrounding the visual display; the panel was illuminated to the same mean luminance as the computer display. Subsequent experiments were conducted without the large surround panel as it was not found to influence the data.

The experiments were controlled by Monkeylogic software (Asaad et al., 2013). A Cerebus recording system (Blackrock Microsystems) recorded LFPs and spiking activity at 30 kHz. RFs, defined as minimum response fields, were hand-mapped, with small bars of light, based on spiking activity. All RFs were located in the lower left visual field; the average eccentricities of RFs in the two animals were 3.6 deg (SD = 0.9 deg) in Monkey F and 4.5 deg (SD = 0.5 deg) in Monkey S. Histological reconstructions have not been made as the animals are involved in ongoing follow-up experiments. Based on electrode length, RF organization, and tuning properties, the neurons studied were most likely in cortical layer 3.

The position of the right eye was recorded at 2 kHz using an EyeLink 1000 infrared eye tracker (SR Research). Calibrated eye position was continuously reported to the Monkeylogic software and saved by the Cerebus recording system for offline analysis. A photodiode connected to the Cerebus hardware was used to confirm the timing and duration of fixation points relative to neural activity.

The data analyzed came from three data sets, two in animal F (F1, F2) and one in animal S. The F1 and F2 data sets came from different recording arrays that were implanted 8 months apart at slightly offset cortical locations. Data set F1 consists of recordings on 16 electrodes and data set F2 comes from 24 electrodes (the channels that were functional). Data in the S data set come from all 96 electrodes on the array.

### Experimental Design

Each trial began with the illumination of 1 of 12 possible fixation points. These initial fixation points were spaced in 30 deg increments around a circle; the circle had a radius of 7-deg visual angle and was centered on the display. The only stimulus in the RFs under study was the uniform gray background of the visual display. For a trial to continue, an animal had to acquire the fixation point within 3 s (1 deg fixation window). After fixating for 400 ms, the first fixation point was extinguished and a second fixation point at the display center (center of stimulus circle) was illuminated. The animal made a 7-deg saccade to acquire the center fixation point; the trial was kept if the second fixation point was acquired within 250 ms (typical saccade latency was 130 ms). Successful trials required that the central fixation point be held for at least 200 ms, at which time the fixation point was turned off and a liquid reward given. In all cases, the only stimulus in the RF during the saccade and on the two fixations was the dim uniform gray background. The task was an outside-in saccade so that the critical measurements of peri-saccadic neural activity were always made under identical conditions regardless of the location of the initial fixation point and the direction of the saccade. An inside-out version of the experiment was also run in which saccades were made from the display center to 12 points on an imaginary circle around the center. The two versions of the experiment gave comparable results; the outside-in version was used exclusively in the analysis presented here.

The onset and end of the saccade on each correct trial were determined using a velocity-based algorithm (Smeets and Hooge, 2003) and confirmed by visual inspection. To establish the beginning and end of a saccade, the mean and standard deviation of eye velocity were computed during steady fixation at the first fixation point. The onset (end) of the saccade was defined as the time at which eye velocity first exceeded (fell below) 3 standard deviations of the fixation velocity mean. The end of a saccade, and thus the start of the subsequent fixation, was marked as time *t* = 0.

### Data Analysis

**Figure 1:**
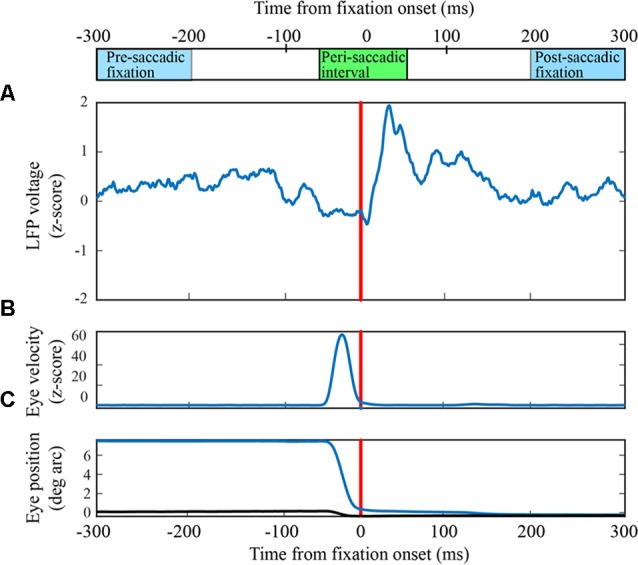
Peri-saccadic signals in primary visual cortex (V1) from the F1 data set. **(A)** Local field potential (LFP) on a single electrode averaged over 63 leftward saccades. **(B)** Average eye velocity. **(C)** Average horizontal (blue) and vertical (black) eye position.

Analysis was conducted on 1775 trials. Each of the three data sets had trials in 12 different directions (62–63 trials per direction in F1, and 42–44 trials per direction in F2 and S). The numbers of trials were not always identical across directions because trials were discarded in *post hoc* analysis if a corrective saccade was made. LFPs were obtained by low pass filtering at 300 Hz and then down-sampling the 30 kHz raw data at 1 kHz. Data were analyzed in a variety of temporal windows identified by either the start and end times of the window or the window center (τ). The time period −50 to +49 ms (the *τ* = 0 interval) was defined as the peri-saccadic interval (Figure 1; *t* = 0 marks saccade end); this interval extended into the post-saccadic fixation in case the early portion of the fixation LFP carried information about the saccade. The intervals −300 to −201 ms (the *τ* = −250 interval) and +200 to +299 ms (*τ* = +250) were defined as the pre-saccadic and post-saccadic fixation periods, respectively. *Post hoc* analysis confirmed that there were no saccades in these fixation intervals. The saccade and fixation time periods were usually represented by LFP samples of length 100 ms (at 1 kHz).

### Support Vector Machine (SVM)

Of the many methods that might be used to classify fixation and peri-saccadic LFPs, we used a linear SVM because it is a simple agnostic data-driven approach that is readily interpreted. Distinct SVM classifiers were constructed for each LFP channel. Prior to training the SVM, data were combined across all trials to compute a mean and standard deviation of the recorded LFP voltages for each channel. On each trial, the mean voltage was subtracted from each channel’s LFP and the LFP was normalized by dividing by the standard deviation. A classifier was built using the normalized training data set, with a linear kernel and 100 unweighted features corresponding to 1 ms samples of the 100 ms training data in the pre-saccadic, peri-saccadic, or post-saccadic time interval. The positive class was usually defined as LFP activity in the peri-saccadic interval and the negative class came from one of the fixation intervals. We solved for the maximum-margin hyperplane between saccade and fixation data using sequential minimal optimization in Matlab.

### Assessment of SVM Performance

By definition, the hyperplane, derived by the SVM, maximizes the margin between support vectors of the positive and negative classes. One way to describe classifier performance is to calculate a percent of correct classifications based on this hyperplane. However, the SVM hyperplane does not, by design, lead to the maximum percent correct and it does not distinguish Type 1 and Type 2 classification errors. We chose to use a different approach to quantify classifier performance that builds on the SVM hyperplane, but is not tied to the SVM criterion or any other particular criterion. We start with the SVM hyperplane and quantify the trial-by-trial distance of vectors in the test data from the separating hyperplane of the training set. The vector distance is referred to as the “s-value” (i.e., the signed distance to the hyperplane). Let *H*^(k)^ represent the training set obtained by removing the k’th training trial. An SVM was trained on *H*^(k)^; and a hyperplane, H, defined. Then s^(k)^ is the signed distance of the k’th training example to H. Using this leave-one-out cross validation across all values of k (i.e., all trials), two histograms of s-values were constructed, one for saccades and another for fixations. A positive class s-value is a metric of the likelihood that a vector belongs to the (positive) saccade class.

A receiver operating characteristic (ROC) was constructed by varying the threshold (i.e., hyperplane) for positive (saccade) classifications (i.e., a range of classification hyperplanes was used, all parallel to the SVM hyperplane). With each threshold, the true positive rate (TPR) and the false positive rate (FPR) were recorded (i.e., unlike % correct, Type 1 and Type 2 classification errors were distinguished). Plotting the TPR and FPR on the vertical and horizontal axes, respectively, generated a ROC. The area under the ROC curve (AUC) is the probability that a random positive example has a higher s-value than a random negative example. In this way, AUC is an overall measure of classification performance that is not tied to any particular criterion.

### Temporal Aspects of Classification

Of particular interest in this study was the accuracy of the temporal information about fixations and saccades carried in the LFP. We examined this point by varying the times of both the training and testing SVM windows. As above, leave-one-out cross validation was used, We first constructed a family of SVMs trained using 100 ms temporal windows with *τ*_train_ ∈ {−50,−49,‥.,49} used to define the positive class. We then classified 100 ms segments of test LFP data, one trial at a time, to determine which value of *τ*_test_ gave the largest s-value (i.e., the best classification performance). Note that if the range of *τ*_test_ was fixed at {−50, 49}, there would be bias in the results. For example, with *τ*_train_ = −50 ms, the “best fit” test data would be forced to have *τ*_test_ ≥ −50 ms which would lead to a significant mean error. To determine which *τ*_test_ gave the largest s-value, without bias, it was necessary to extend the range of test windows following two rules: (1) the range of *τ*_test_ values was symmetrical about *τ*_train_; (2) the range of *τ*_test_ was chosen so that, for any value of *τ*_train_, *τ*_test_, spanned the range {−50, 49}. Thus, if *τ*_train_ is the center of a 100 ms training window, we computed s-values using test data with centers *τ*_test_ ∈ {*τ*_train_ − 100, *τ*_train_ − 99, ‥., *τ*_train_ + 99}. This allowed us to test the hypothesis that classification is best when *τ*_test_ = *τ*_train_. To the extent that this is true, the LFP could potentially be used to accurately infer the timing of fixation onset or some other peri-saccadic event. The *τ*_test_ that gave the largest s-value, was taken as a measure of an SVMs “vote” on a given trial (i.e., the segment of the test data LFP that was most similar to the training data LFP; see Figure 4A). To quantify the extent to which *τ*_test_ = *τ*_train_ yielded the best saccade/fixation classification, we computed the RMS error for each value of *τ*_train_ (e.g., Figure 4C).

**Figure 2:**
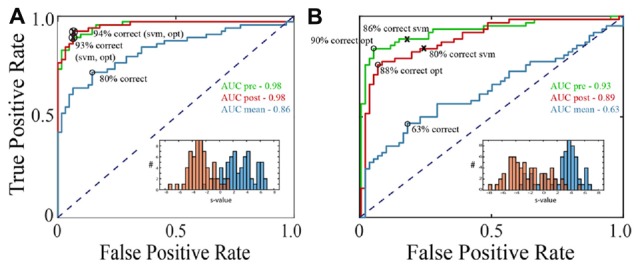
Receiver operating characteristic (ROC) curves for classification of LFPs as fixation or saccade. **(A)** Classification with an exemplary channel from the F1 data set when the negative (fixation) class was based on the pre-saccadic fixation (green) or the post-saccadic fixation (red). Performance was comparable with either definition of the negative class (AUCs in upper right corner). The inset shows the frequency of s-values when the negative class (red histogram bars) was defined as the post-saccadic fixation. The positive (peri-saccadic) class was defined as −25 to +75 ms relative to fixation onset (blue histogram bars). Also shown are percent correct classifications based on the support vector machine (SVM) hyperplane (svm) and an “optimal” criterion that assumes the cost of false positive and false negative errors are the same (opt). For this channel, these two criteria gave the same percents correct. Finally, the blue curve shows classification performance using a simple measure of mean LFP amplitude in a 100 ms epoch; performance was significantly lower than with the 100-dimensional SVM classifier based on % correct and AUC. **(B)** The same performance measures as in **(A)** for a different F1 channel. In this case, the optimal criterion gave higher percents correct than the SVM criterion and the AUC based on the pre-saccadic fixation was somewhat better than the post-saccadic fixation. Again, classification (AUC) was significantly reduced using the mean peri-saccadic LFP amplitude rather than the 100-dimensional LFP shape.

**Figure 3:**
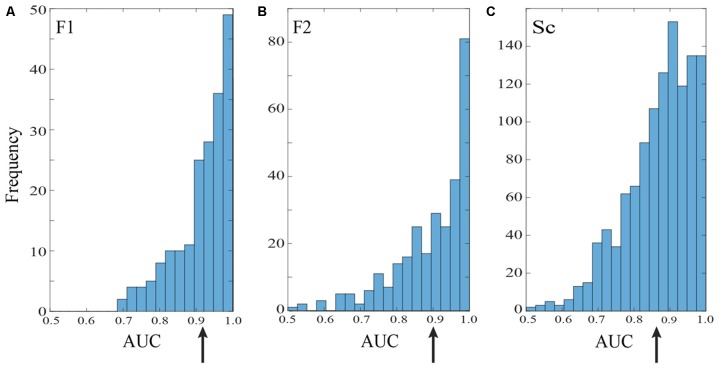
Saccade vs. fixation classification across recording channels in the three data sets. Histograms show the frequency of AUC values with separate entries for each saccade direction; areas under the ROC curves were generally well above chance for all three data sets. Mean AUCs (arrows) were 0.92 for F1 **(A)** 0.90 for F2 **(B)** and 0.87 for S **(C)**.

**Figure 4:**
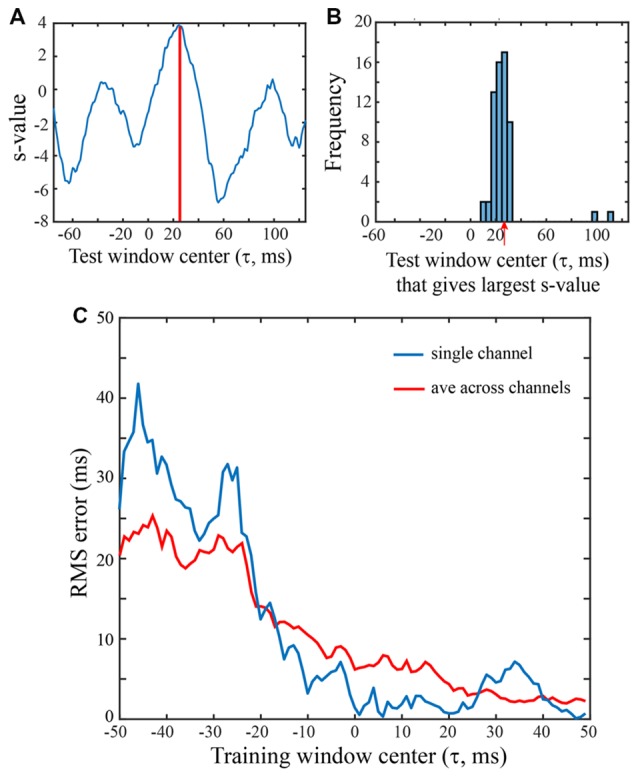
Saccade vs. fixation classification using a range of LFP temporal windows. All SVMs were trained on 100 ms of LFP data from a single channel of the F1 data set. Saccades were in the 0 degree direction. **(A)** Classification of an LFP on a single trial, using test data from a range of temporal windows. An SVM was trained on LFP data with the saccade epoch defined as the *τ* = 25 ms temporal window and the fixation epoch defined as *τ* = 25 ms temporal window and the fixation epoch defined as*τ* = 200–299 ms. The classifier (hyperplane) constructed for this SVM was then used to classify peri-saccadic LFP data from 200 different (100 ms) temporal test windows, centered from −75 ms to +124 ms. With each of the test windows, the peri-saccadic data define a point in 100-dimensional feature space and the distance of the point from the *τ* = 25 ms SVM classifier hyperplane (margin) defines the s-value. Here, the s-value is largest, and the classification performance best, with a test window at *τ* = 25 ms, matching the “correct” (training) value. **(B)** Distribution of “best” test-window centers (i.e., corresponding to largest s-values) for 62 saccades in the 60 deg direction. The distribution peaks at *t* = 26.2 ms, close to the “correct” time that the SVM was trained on. **(C)** RMS error was computed from the s-value “votes” when the training window ranged from −50 ms to 49 ms. Based on early LFP training (i.e., left side of graph), RMS errors were about 20–30 ms. As the training window moved rightward beyond −20 ms, the error dropped significantly, eventually reaching values below 5 ms.

### Classification Based on Pre-saccadic vs. Post-saccadic Fixation Period

Peri-saccadic LFP activity was compared with LFPs recorded during two different periods of fixation, the pre-saccadic fixation and the post-saccadic fixation. Pre-saccadic fixation activity came from the time period −300 to −201 ms relative to the start of a fixation. Post-saccadic fixation activity was defined as 200–299 ms relative to fixation start. A comparison with the post-saccadic fixation provides the tightest control over possible visual stimulation variables because, regardless of the location of the previous fixation and the direction of the saccade, the critical comparison is with LFPs on a final fixation that was always at the same display location. The reason we also made comparisons with pre-saccadic neural activity is that it may reflect the analysis challenge faced by the brain. For example, it might be important that the brain extracts information from the LFP concerning the end of a saccade and start time of a fixation. This might be accomplished by monitoring the LFP for a pattern that distinguishes it from a previous fixation pattern.

### Significance of Variations in LFP Shape Across Saccade Direction

A permutation test was used to assess the significance of variations in LFP shape that were observed when saccades were made in different directions. For each of 12 saccade directions, an overall within-direction Pearson correlation coefficient was computed as the average of the correlation coefficients obtained with all possible combinations (across trials) of the peri-saccadic LFPs (τ = 25 ms) in that direction. Shuffled correlation coefficients were also computed for all possible pairs of directions. To do this, the LFPs for two directions were pooled and half randomly assigned to each of the direction labels. Average correlation coefficients were separately computed for each of the two groups and the average of these two numbers constituted a sample. This process was repeated for 10,000 permutations to yield a distribution of correlation coefficients for the direction pair. A *p*-value was obtained, for a pair of directions, by comparing the average of the two within-direction correlations with the distribution of shuffled correlation coefficients for that pair.

#### Direction Sensitivity

To assess the sensitivity of V1 LFPs to saccades in different directions, we calculated saccade/fixation classification performance separately for each of 12 saccade directions spaced in 30 deg increments. Thus, 12 ROC curves were constructed for each of the recorded channels. Classification performance in a particular direction, across all the channels studied on an array, was quantified by summing the positive-class scores (s-values) from all the channels and constructing an overall ROC (associated AUCs in Figures 7, 8). The family of 12 ROC curves, for a particular data set, shows how classification performance varies with saccade direction.

**Figure 5:**
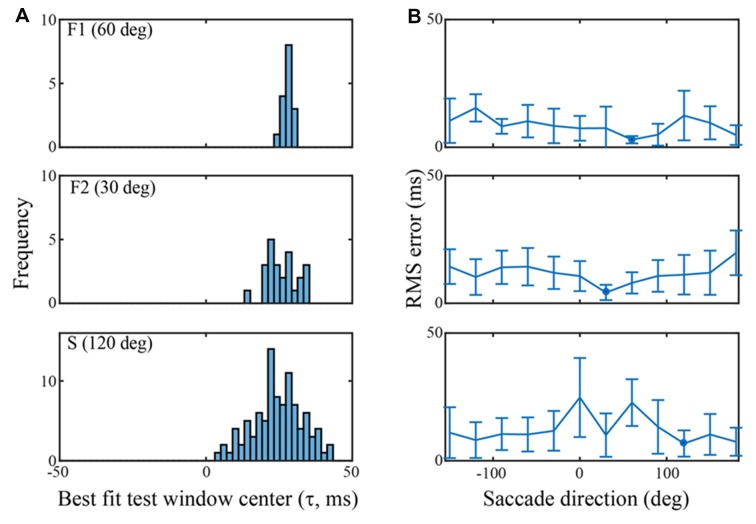
Classification performance across channels in each of the three data sets using an SVM trained on the τ = 25 ms peri-saccadic LFP. **(A)** Distribution of best test-window-centers for every channel in each of the data sets. The distributions for the F1, F2, and S data sets come from saccades in the 60, 30, and 120 deg directions, respectively. The distribution means are near τ = 25 ms (F1: 28.0; F2: 25.8; S: 24.4 ms). **(B)** RMS errors of the best test-window-centers (i.e., relative to τ = 25 ms), in each of 12 saccade directions. The blue spots indicate the means of the distributions in **(A)** Values near 0 ms RMS error indicate classification that was best when the LFP test window matched the training window.

**Figure 6:**
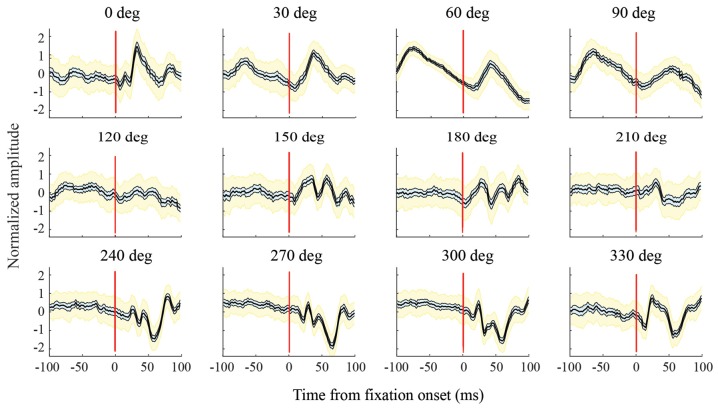
Variations in LFPs with saccade direction. For this channel, in the F1 data set, and all the other channels analyzed, LFPs in a given saccade direction had a consistent shape across trials. The bold black line in each graph shows the mean LFP across saccades; thinner lines to the sides of the mean (and cyan shading) show the 95% confidence intervals calculated using a bootstrap procedure; yellow shading shows standard deviations. As saccade direction changes, there are sequential directions with similar LFP shapes, but across a full 360 deg, there are large variations in LFP shape. For all combinations of saccade directions, differences in LFP shape (internal correlations) were significant at the *p* = 0.05 level.

**Figure 7:**
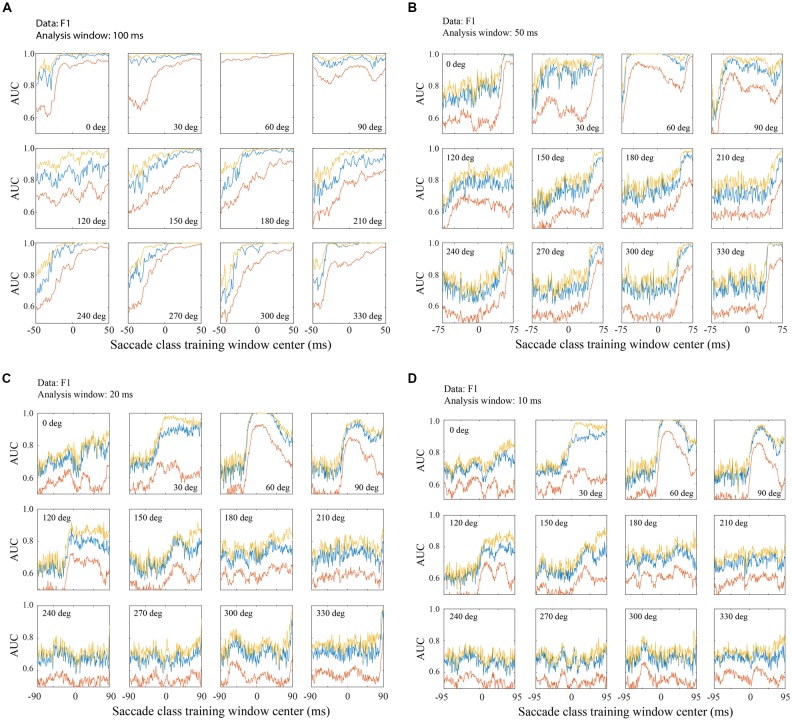
Classification across saccade directions with the F1 data set. **(A)** Performance with 100 ms training and testing windows. An SVM was trained with fixation defined as 200–299 ms relative to fixation onset and a saccade class centered on the time indicated on the horizontal axis. Each panel represents a different saccade direction, as indicated. Classifier performance was quantified using test LFP data from a temporal window matching the saccade class training window. The red line shows AUC averaged across all channels, the blue line is from the single best channel, and the yellow line is the highest AUC obtained by sequential addition of channels. Figure parts **(B–D)** follow the same conventions as **(A)** but use training and testing windows of 50 ms **(B)** 20 ms **(C)** and 10 ms **(D)**. Classification performance is better with later training/testing windows of time, but performance is often above chance even with windows that are entirely pre-saccadic. Not surprisingly, performance is better with LFPs in longer temporal windows, but even with only 10 ms snippets of LFP data, classification was often well above chance. Note that the overall range of times analyzed, with each window duration, was −100 to 100 ms relative to saccade end. Thus, as the training window duration decreased **(A–D)**, the training window centers spanned a larger range of time (e.g., a 100 ms window centered at τ = −50 begins at −100 ms and a 10 ms window centered at τ = −95 ms also starts at −100 ms).

**Figure 8:**
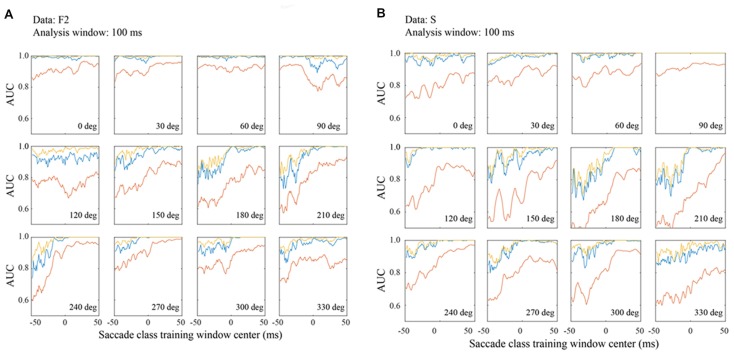
Classification performance in the F2 **(A)** and S **(B)** data sets with matched 100 ms training and testing windows. Figure conventions are the same as Figure 7.

We also used SVM classifiers constructed for each of 12 saccade directions to determine the accuracy with which LFPs might be used to infer saccade direction. Training and testing data came from the τ = 25 (−25 to +74 ms) window relative to fixation onset as this time period generally yielded high AUCs. First, we constructed one-direction vs. all-other-direction classifiers. Using leave-one-out cross validation, data on every trial but one were located in 100-dimensional feature space and 12 classifiers were constructed. That is, for each of the 12 saccade directions, a hyperplane was defined that distinguished data for saccades in one direction (positive class) from all the other saccade directions (negative class). An AUC was computed that represented the likelihood that a randomly drawn example from the positive class had a larger s-value than a randomly-drawn example from the negative class. This procedure was repeated for each channel. Additionally, a 12-way SVM, across saccade directions, was constructed using fitecoc() in Matlab. In this analysis, all possible pairwise SVMs were constructed and used to define the 12-way classifier. Because of the computation time required, this analysis used 30-fold cross validation rather than leave-one-out. The one-direction-vs all-other-direction classifier and the 12-way classifier analyses are used in Figure 10.

**Figure 9:**
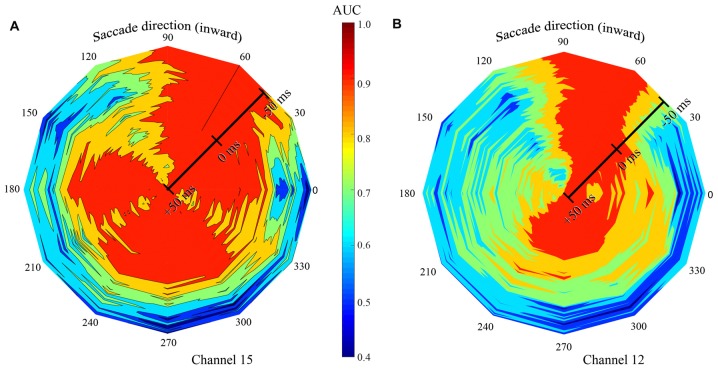
Summary of classification performance (AUC) across time and saccade direction for two exemplary channels in the F1 data set. Low AUCs are blue to green and high AUCS are yellow to red. Color was linearly interpolated between data from the 12 saccade directions. Saccade direction is indicated “around the clock” and the center (τ) of the 100 ms training and testing window is shown radially. **(A)** The signal recorded on channel 15 gave best classification performance, with all LFP time windows, with saccade directions of 60 and 90 deg. High AUCs were also achieved at a broad range of other saccade directions, but only with later temporal windows. **(B)** The LFP on channel 12 yielded high performance only with the 60-, 90-, and 270-deg saccade directions.

**Figure 10:**
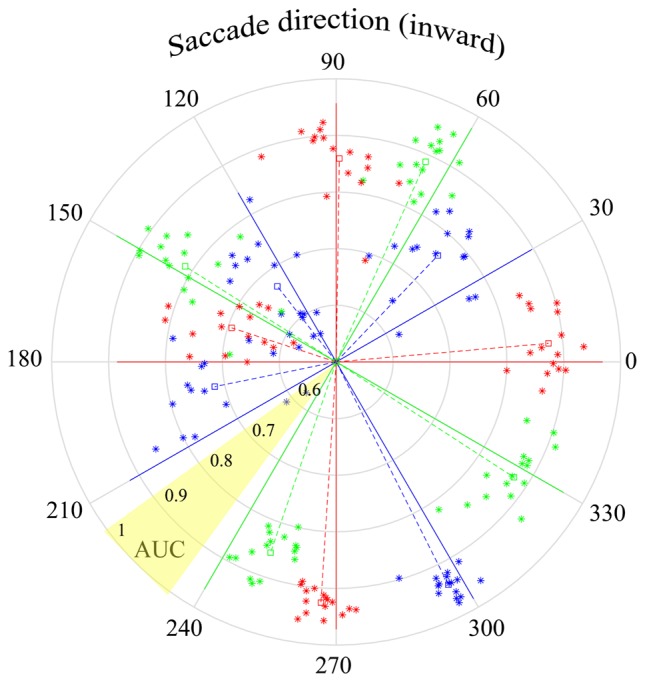
Peri-saccadic LFPs used to infer saccade direction. The τ = 25 ms time epoch was used with the F1 data set. Solid lines in each of the 12 saccade directions show the AUC for classification in the correct direction vs. all other directions (based on an optimal combination of channels). The star symbols show performance separately for each of the 16 F1 channels, where the radial distance from the origin shows the one-direction vs. all-other-directions AUC for that channel. The radial distance of the dashed lines shows AUC averaged across channels. The polar angles of the symbols and dashed lines are the direction votes, averaged across trials, based on a 12-way saccade-direction classifier. The angular separation between the dashed and solid lines of a given color indicates the accuracy of the inferred saccade direction. As indicated in the yellow shaded sector, AUC increases from 0.5 at the center to 1.0 at the outer edge of the circle.

#### Single Channel and Population Performance

Classification performance is presented for LFP data combined several ways across channels. For example, comparisons are made between the single best channel (i.e., the one that gave the highest AUC) and the average of all channels. We also estimated performance based on a subset of the channels analyzed. This was done in a progressive manner starting with the single best channel and sequentially adding additional channels in order of descending AUC. With more than one channel, histograms were made that pooled s-values (distances from SVM separating hyperplane) across channels for fixation and peri-saccadic time periods. Performance of the population was quantified as the AUC of the associated ROC function. It was generally found that as the 2nd and 3rd best channels were added, performance improved but at some point, adding more channels degraded performance as the fixation and saccade histograms of s-values became more overlapped. “Optimal performance” is defined as the highest AUC obtained by the sequential addition of channels and we refer to these channels as the “optimal combination.”

## Results

Eye-movement related signals in area V1 were generally visible when LFPs were averaged after aligning neural activity on saccade end (fixation onset). As the saccades used in this analysis were of fixed length, the average LFPs were very similar when they were aligned to saccade onset. We found that the clarity and consistency of the saccade-related signals were variable across electrodes and animals: in some cases, inflections in the LFP were reliably associated with saccades on individual trials and in other cases the correlation became apparent only after averaging across many saccades. Figure 1A shows the average LFP from one channel in the F1 data set along with the average horizontal eye trace over 63 leftward saccades (blue curve in Figure 1C). Fixation onset was defined by the return of eye velocity to less than 3 standard deviations away from the fixation mean (Figure 1B). The average LFP is relatively flat up to the start of the new fixation at which time there is a small dip followed by a peak around 30–40 ms, and a second dip around 60–70 ms. This pattern is similar, though not identical, to reports in previous studies (Rajkai et al., 2008; Ito et al., 2011). Variations in the LFP, across recording channels and saccade directions, are discussed below.

### Classification of LFPs as Fixations or Saccades

The first question we investigated was the extent to which a SVM can classify 100 ms epochs of the LFP as a period of steady fixation or a period in which a saccade occurred. We performed this analysis on the three data sets from two animals. With the F1 data set we trained two different SVMs; in one the negative (fixation) class came from a pre-saccadic fixation interval and, in the other, the negative class used a post-saccadic fixation interval. In both SVMs, the positive (saccade) class was trained on data in the peri-saccadic window −25 to +74 ms (τ = 25 ms). The negative class in the first SVM came from the interval −300 to −201 ms (τ = −250 ms) and the second SVM used the post-saccadic interval 200–299 ms (τ = 250 ms). Testing the performance of an SVM consisted of measuring, on each trial, the distance (s-values) of 100-dimensional vectors (100 ms LFP data), taken from the negative and positive class temporal epochs, from the separating hyperplane. Separate frequency distributions of the s-values were made for data coming from the fixation and saccade intervals.

Using data from leftward horizontal saccades in two F1 channels, Figure 2 shows ROC curves constructed by varying the threshold for classification. The green traces are ROCs using a pre-saccadic fixation for the negative class and the red traces are ROCs using a post-saccadic fixation for the negative class. In Figure 2A, the AUC was 0.98 using either the pre- or post-saccadic negative class. In Figure 2B, AUC = 0.93 with a pre-saccadic negative class and 0.89 with a post-saccadic negative class. ROC analysis of single channel data from the F2 and S data sets also showed comparable performance using the pre-saccadic and post-saccadic fixation intervals. As the choice of fixation epoch did not significantly affect the results, in all subsequent figures we only show data using the post-saccadic fixation comparison. There is greater control with the post-saccadic fixation in the sense that the final fixation location, and thus any conceivable visual input, was always the same.

For comparison, Figure 2 also shows other measures of classification performance. The “x” symbols show the percentage of correct classifications based on the hyperplane chosen by the SVM; the circle symbols show the “optimal” percent correct assuming equal cost for false positive and false negative errors. In Figure 2A, the percent correct measures were similar using either the SVM or optimal criteria; in Figure 2B, the optimal criterion gave higher percents correct than the SVM criterion. The percents correct using either criterion was approximately correlated with the area under the ROC. As mentioned in Methods, the figures in the rest of the Results use AUC because it provides an overall description of classifier performance and it does not rely on any particular criterion.

The traces in Figure 1 show that, in addition to inflections in the LFP, there is an increase in mean LFP amplitude in the peri-saccadic interval. This raises the question whether the saccade/fixation classification performance we quantified is based on the shape of the LFP or simply changes in mean amplitude in the peri-saccadic time period. To investigate this question, we first computed mean LFP amplitudes in the peri-saccadic and fixation temporal intervals for the same leftward saccade trials used in the 100-dimensional SVM above. ROC curves were constructed by moving a threshold across the LFP amplitude distributions (peri-saccade and fixation) and calculating the true positive and false positive rates. For the two channels shown in Figure 2, the AUC values were significantly higher with the 100-dimensional SVM than the simple amplitude classifier (Figure 2A: SVM −0.98, mean amplitude −0.86; Figure 2B: SVM −0.93, 0.89, mean amplitude −0.63). As an additional check, we ran the classifier after subtracting the LFP mean from the raw LFP. Interestingly, classification performance using this “shape-only” LFP was almost identical to performance when the mean was not subtracted (because they were similar to the ROC curves using the full LFP, the shape-only curves are not shown in Figure 2). We concluded that classification was generally significantly better using the shape of the LFP in the SVM (shape-only or shape plus mean) rather than simply the LFP amplitude in 100 ms time epochs. Moreover, it appears that most of the information available in the LFP mean amplitude is redundant with information in the LFP shape. We discuss the significance of LFP shape below in the context of direction sensitivity.

Figure 3 summarizes the classification performance across all channels examined in the F1, F2, and S data sets with a training window of τ = 25 ms. The histograms tally AUC values separately for each of 12 saccade directions on each channel. In all three data sets, the majority of channels and saccade directions gave AUCs between 0.9 and 1. The mean AUCs for the data sets were 0.92, 0.90, and 0.87 for F1, F2, and S, respectively. We conclude from this analysis that LFPs in area V1 generally provide information that can be used to reliably distinguish time periods containing saccades and fixations.

### Temporal Accuracy of Classification Performance

We next examined the temporal accuracy with which fixation vs. saccade classifications could be made. This might be critical, for example, if the brain were to use LFPs in V1 to infer fixation onset time in order to enhance processing at the start of each fixation. To address questions about the temporal aspects of classification, we constructed a family of SVMs, trained on data from a range of temporal intervals. The negative (fixation) class always came from data in the 200–299 ms (τ = 250 ms) time period. Different SVMs were trained with the positive (saccade) class defined as a 100 ms window ranging from τ = −50 to +49 ms. As detailed in Methods, with each of these definitions of the “saccade” class LFP, we quantified classification performance using a wide range of 100 ms duration test epochs of LFP data (using leave-one-out cross validation). Each trial contributed a vote based on which τ_test_ gave the largest s-value; i.e., which patch of the test LFP data was most similar to the training segment of the LFP. Based on votes across all trails, the RMS error was computed, an indication of the extent to which the best classification performance was obtained with τ_test_ = τ_train_.

Figure 4A shows s-values obtained from a single trial on one F1 channel when the SVM was trained with τ = +25 ms and test data came from 200 windows with τ ranging from −75 ms to +124 ms. In this particular trial, the largest s-value was obtained with test data having τ = 25 ms. The histogram in Figure 4B was constructed by counting the optimal (“best fit”) test windows (τ values) across all trials for this F1 channel. The mean of the histogram in Figure 4B is 26.2 ms which indicates that when the saccade class was trained with LFPs centered on 25 ms, the best saccade/fixation classification was obtained with test LFP data from approximately the same temporal interval. The temporal match between train and test LFPs could conceivably be used by the system to infer the timing of oculomotor events. For example, by monitoring the LFP over time for a pattern similar to a known peri-saccadic pattern, the system could infer when a saccade occurred, and a new fixation began (see Figure 5).

Figure 4C summarizes the RMS error obtained from the s-value “votes” using training windows ranging from τ = −50 to +49 ms; there are two curves, one for a single channel (blue) and the other averaged across the 16 F1 channels (red). To the left side of the plot, with τ values before the start of the saccade, errors are around 20 ms (saccade start was approximately *t* = −30 ms). Roughly around τ = −20 ms, the error drops and progressively declines as τ increases to more positive values, error ultimately reaching below 5 ms. The single channel has larger errors in earlier time epochs but reaches quite low values (1–3 ms) at many of the τ values above zero. The best classification performance (lowest RMS error) clearly comes from LFP segments in the peri-saccadic to post-saccadic intervals, though even at early times, the error is only 20 ms. We conclude that there is sufficient information in the LFP to precisely infer the start of a new fixation. Whether the brain could rapidly make use of this information to determine the occurrence of a new fixation in real time requires different analyses which we are pursuing. While some channels conveyed more precise temporal information than others, even the average LFP gave significant timing information. The performance similarity with different individual-channel LFPs suggests that oculomotor information is ubiquitous in the area of cerebral cortex underlying the 4 × 4 mm recording arrays. Looking toward further analysis below, we note that classification performance has a complex dependence on the location of the temporal analysis window, the duration of the temporal analysis window, the saccade direction, and the channel(s) under study.

Figure 5 summarizes classification performance for each of the three data sets using the τ = 25 ms training window. Figure 5A provides a summary across channels, for each data set, using a saccade direction that gave good classification performance (F1: 60 deg, F2: 30 deg, S: 120 deg). The frequency distributions in Figure 5A show the test window center, τ that, on average, gave the largest s-values on each channel when the training window was τ = 25 ms (i.e., the histogram is constructed from values as indicated, for one channel, by the red arrow in Figure 4B). For the F1, F2, and S data shown, the means of the distributions are 28.0 (SD = 1.4), 25.8 (SD = 5.2), and 24.5 (SD = 8.5) ms, respectively. Thus, the error in the average s-value “votes” was 1–3 ms on average. Figure 5B shows the RMS error, averaged across all channels, in each of the 12 saccade directions. The circular blue symbols show the error using the saccade direction indicated in Figure 5A. There was some variation in the RMS error with saccade direction, but this was insignificant relative to the standard deviation across channels (error bars). The mean RMS errors across all channels and all saccade directions was 8.5 ms (SD = 6.9), 11.8 ms (SD = 7.5), and 12.2 ms (SD = 10.3), for the F1, F2, and S data sets, respectively. Comparing Figures 4 and 5 we conclude that there is significant temporal information about saccades and fixations even when all channels are combined. That said, temporal precision varies considerably across channels, some channels carrying quite precise information and others less so. If an optimal(s) channel is used, it is possible to monitor the temporally changing LFP and establish the onset of each new fixation within a few milliseconds based on the s-value; if the average across channels is used, the error increases.

### Directional Sensitivity, Temporal Window Size, and Pooling Across Channels

The results presented so far have concerned the ability to use peri-saccadic LFPs to infer the timing of saccades and fixations. Additionally, we were interested in determining whether LFPs are sensitive to saccade direction. Animals made 7-deg saccades from 12 different start locations, spaced in 30 deg increments around a circle. All saccades were made to the same end point in the center of the display.

Figure 6 shows LFPs from an exemplary channel in the F1 data set. These are normalized peri-saccadic field potentials (τ = 25 ms) recorded in each of the 12 saccade directions. The bold central curve in each graph shows LFPs averaged over all saccades with the same direction. LFP shape was generally consistent in each saccade direction; the thin curves (and cyan shading), to each side of the average, show 95% confidence intervals of the average LFP and the yellow shading shows LFP standard deviation. There was often a similarity in LFP shape over a limited range of neighboring saccade directions; for example, 0–30 deg and 240–300 deg. However, over the full 360 deg range of saccade directions, there were large variations in LFP shape. We found high classification performance (AUC values) with diverse LFPs that had unimodal, bimodal, and more complex shapes. To assess the significance of differences in LFP shape, we performed a correlation analysis (Methods) to test the null hypothesis that LFPs recorded with different saccade directions actually came from the same distribution. For the channel shown in Figure 6, all *p* values were less than 0.05. Of the *p*-values obtained in this way (Bonferroni corrected), across all channels, 97.5% were less than 0.05 and 96.2% were less than 0.01. This indicates that virtually all pairs of saccade directions had significantly different LFPs. As classification performance was high across directions (see below), it does not appear that there is a single peri-saccadic LFP shape that signals the occurrence of a saccade and/or fixation.

Figure 7 shows classification performance (area under the ROC) across the 12 saccade directions, using the F1 data set. The negative (fixation) class was trained on the post-saccadic fixation epoch (200–299 ms relative to fixation onset). Figure 7A shows AUCs obtained when the positive class (saccade) was trained on a range of 100 ms duration peri-saccadic windows as indicated on the horizontal axis (τ = −50 to 49 ms). Testing always used the same temporal window as the positive class training window. By making recordings with many electrodes simultaneously, we were able to assess the trial-to-trial performance with individual channels and combinations of channels. The red line shows the AUC averaged across all channels, the blue line shows the single best channel, and the yellow line shows the optimal combination of channels as defined in Methods. It should be noted that in Figures 7, 8 the single best channel and the optimal combination were selected independently for each saccade direction. Thus, across all directions, the blue and yellow curves do not reflect performance of one particular channel nor a specific channel combination.

In general, and regardless of which channels are plotted, performance is lowest with the earliest temporal windows and improves in later windows. Note that the earliest window (τ = −50 ms) starts before saccade onset and ends at the beginning of the new fixation (*t* = 0). Thus any performance above chance at τ = −50 ms is based entirely on LFP activity prior to the fixation. The AUC was above chance, even in this early time window, for all saccade directions and for all channel combinations shown; for the best channels and saccade directions, performance was often quite high based solely on the pre-fixation LFP. Thus, significant information about the occurrence of saccades is available in V1 before and/or during saccades. Conceivably there is eye movement information at even earlier times than those shown in Figures 7, 8; we limited the range of the positive (saccade) class window locations to prevent them from overlapping with the negative (fixation) class window. As the training and testing windows shift to include increasing amounts of post-saccadic times (τ > −50 ms), the AUC increases. In many cases, the AUC saturates at middle time windows and stays at this level through the latest windows plotted. With a 100 ms window, performance generally reaches its peak value with a window center near τ = 0. We conclude from this observation that in the peri-saccadic interval, −50 to 50 ms, the LFP carries considerable information that distinguishes a saccade in any direction from a period of fixation. It is also possible to identify a completed saccade based on an entirely post-saccadic LFP window (τ = 50 ms).

While performance generally approaches saturation levels with saccades in most directions, there was some sensitivity of classification performance to the direction of the saccade. For example, saccades in the 60–120 deg directions were rather insensitive to the timing of the testing window compared to other directions. The 60 deg direction is noteworthy because classification performance was near-perfect based on LFPs in any of the temporal windows used, ranging from entirely pre-fixation to the fixation period after saccade completion. This observation is intriguing because all three data sets were obtained with electrode arrays in the right hemisphere that yielded RFs in the lower left visual field. Therefore, saccades in the 60 deg direction moved the fovea toward the RFs of the recorded units. Further testing is underway to assess the reliability and significance of this finding but, as outlined in the Discussion, it is unlikely that the bias in this direction resulted from visual stimulation. Saccades in the range of approximately 120–210 degrees require later temporal windows to reach saturation than saccades in other directions.

Comparing the three traces in each of the panels in Figure 7A, there is a consistent pattern. When all the channels analyzed are combined (red) performance is not as good as the best channel (blue) or an optimal combination. In later time windows this difference is often minimal (after performance saturation). However, the best channel reaches peak performance in earlier time windows than the average across channels. In most cases the optimal combination of channels (yellow) yielded performance that was only marginally better than the best channel.

Figure 7A suggests that saccade/fixation classifications can be reliably made based on activity during a saccade, during a fixation, or across the peri-saccadic period, but inferences about timing are limited by the 100 ms analysis window size. This raises the question, is it possible to estimate more precisely what LFP times and durations can be used to reliably distinguish saccades and fixations? To address this question, Figures 7B–D show classification performance (AUC) across saccade directions when the analysis window was reduced to 50 ms (Figure 7B), 20 ms (Figure 7C), and 10 ms (Figure 7D).

As the temporal window size is reduced, the saccade directions that give the highest AUCs are unchanged. Predictably, as the temporal window size is shortened from 100 ms to 50, 20, and 10 ms, performance saturates at lower AUC values. That said, perhaps the most striking observation with the shorter window lengths is how similar performance is from 50 ms to 20 and 10 ms. The AUC values are reduced and there is somewhat more variability across test window timing (τ), but overall the AUC patterns are similar. Even with 10–20 ms windows, enough information is extracted from the LFPs to reliably classify saccades and fixations. It is interesting to note that, in the saccade directions that give the largest AUCs, performance sometimes peaks with temporal windows centered about 10–20 ms after the start of new fixations and declines with later windows (e.g., 60 and 90 deg directions in Figures 7C,D). This finding is not consistent enough across saccade directions to be definitive but it hints that a short period of time just after fixation onset might have the greatest “information density” for distinguishing saccade and fixation LFPs.

As window duration is shortened, performance differences across saccade directions and channels become more striking. With a 100 ms window, classification is very high regardless of direction and regardless of whether the best channel or a channel average is analyzed (Figure 7A). With shorter duration windows (Figures 7B–D), performance based on the channel average remains high in a range of optimal directions but is near chance in other directions. On the other hand, if one focuses on the best channels, performance is well above chance in all directions with even the shortest duration LFP windows (Figure 7D).

The temporal placement of the training and testing windows is noteworthy as performance remains above chance even with the earliest and latest temporal windows used. Note that as the windows are reduced in duration, the leftmost window ends earlier and, depending on window duration, longer (or entirely) before the start or end of the saccade. Likewise, the τ = 49 ms window begins longer after the end of the saccade. At the extreme, with a 10 ms window duration, classification AUCs between 0.6 and 0.7 are obtained from the best channels using the earliest LFP window that extends from −100 ms to −90 ms (τ = −95 ms) relative to saccade end. Consistent with other reports (e.g., Freedman, 2008) the 7 deg saccades in our study generally had durations of about 30 ms. This means that classification is possible with a signal that arrives in V1 roughly 60 ms before the saccade begins. At the other extreme, with the τ = 50 ms window, fixation LFPs and LFPs shortly after saccades can be reliably distinguished based on LFPs that are entirely post-saccadic.

Observations derived from Figure 7 suggest that there is a stretch of LFP, from before a saccade begins to after it ends, that can be used to reliably infer the occurrence of a saccade. With even a 10 ms analysis window, performance is well above chance and in certain saccade directions is near perfect. In the best saccade directions, classification performance is very high with selected channels or a channel average; in suboptimal directions performance saturates at moderate levels using selected channels but falls to near chance with the shortest temporal window and suboptimal saccade directions.

To compare performance across the three data sets, Figure 8 shows AUC measurements for the F2 (Figure 8A) and S (Figure 8B) data based on 100 ms temporal windows. In most all regards, the data are comparable to the F1 data shown in Figure 7. For example, performance increases in later temporal windows and usually saturates around τ = 0. Also, the best channel and the optimal combination are similar and significantly better than an average of all channels. In all three data sets, performance is best for a subset of saccade directions, generally 0–90 deg. These are the directions that moved the fovea approximately in the direction of the RFs.

Even more than F1, the F2 data set gives classification performance that is near perfect in all temporal windows when the saccade direction is 0–60 deg. Moreover, even at less optimal directions, AUCs are well above chance at the earliest temporal windows whether one considers the best channel or the channel average. The AUC values for the S data are comparable to those for the other data sets. This suggests that features of the LFP signal that allow it to be classified as fixation or saccade are generic in macaque V1.

Figures 7, 8 show the dependence that classification performance has on the time and duration of the LFP analysis period as well as saccade direction. To convey the richness of the interactions between the time and direction factors, Figure 9 shows classification performance for two channels in the F1 data set where AUC is indicated by color. Saccade direction varies “around the clock” and the temporal window (τ) used for training and testing the saccade class is shown radially. These windows were always matched and 100 ms in duration. The perimeter of the figure corresponds to τ = −50 ms, relative to saccade end, and the figure center shows τ = 50 ms. The asymmetry in the color coding at a fixed radius shows the directional sensitivity of the AUC. Channel 15 (Figure 9A) yields near perfect classification performance in the vicinity of the 60-degree saccade direction irrespective of the LFP analysis window timing (red extending from perimeter to center). Recall that this is the direction that moves the fovea in the direction of the RFs of the recorded neurons. Over a much broader range of saccade directions (all directions except 90–150 deg), the performance measured by AUC is high with later analysis windows (τ values later than about −25 ms) but poor with earlier windows. Taken together these observations indicate that, in an optimal direction, saccade/fixation classifications can be made perfectly based even on an LFP window that ends before the new fixation begins. In other directions, high AUCs are obtained only if later, fixation, portions of the LFP are included. Finally, in a narrow range of saccade directions (90–150 deg) the channel 15 data give low to moderate AUCs regardless of the timing of the LFPs analyzed.

Figure 9B (channel 12) shows high AUCs with saccades in the 60 and 90 deg directions with any analysis time period. In directions rotated 180 deg from optimal (240–270 deg), performance is high if analysis includes part of the fixation period, but the AUCs are much lower using earlier time windows. In comparison to channel 15 (Figure 9A), channel 12 performance falls off much more rapidly away from the optimal saccade directions. Across the other channels in the F1, F2, and S data sets, there was significant variation in similar plots, indicating considerable complexity in the dependence of AUC performance on the time period of the LFP and the direction of the saccade. In other words, there is no single peri-saccadic time at which V1 appears to carry the most information about saccades and fixations; as Figures 7, 8 show, there is a range of “best times” that depends on multiple factors.

### Estimating Saccade Direction

In the analysis above, it was found that the quality of saccade/fixation classification varied with saccade direction. Here we investigate a distinct question: can the LFP be used to infer saccade direction? Instead of saccade/fixation classifiers, we constructed binary classifiers that distinguished different saccade directions. One approach used was the construction of one-direction vs. all-other-direction classifiers. Twelve classifiers were constructed, using leave-one-out cross validation. That is, for each of the 12 saccade directions, a hyperplane was defined that distinguished data for saccades in one direction from all the other saccade directions. An AUC was computed that represented the likelihood that a saccade in a particular direction would be correctly classified as being in that direction rather than any other direction. The solid lines in Figure 10 have polar angles corresponding to the 12 saccade directions; the lengths of the solid lines show the AUC obtained with a combination of channels (optimal based on sequential addition of channels). The AUC values show that optimal direction classifications, based on one-direction vs. all-other-direction classifiers, can be made with high reliability.

For each of the 12 directions in Figure 10, there are 16 star symbols corresponding to the 16 channels in the F1 data set. The radial distance of a symbol from the origin shows the one-direction vs. all-other-directions AUC for that channel. These single-channel measures range between 0.7 and 1.0 but are generally near 0.9. The radial length of the dashed lines indicates the AUC in each saccade direction, averaged across all channels.

The polar angles of the star symbols and the dashed line come from a separate 12-way classification. On each trial, the classifier determined which saccade direction was most likely to have produced the peri-saccadic LFP. For each electrode channel, these direction votes were averaged across all trials that had the same actual test data saccade direction. The direction vote, across trials, is indicated by the polar angle of the star symbols. The polar angle of the dashed line shows the direction vote averaged over all trials and all channels.

For example, based on LFP data collected with saccades in the 90 deg direction, the average estimated saccade direction was 89.1 deg (the dashed and solid red lines are nearly superimposed). On the other hand, the red dashed and solid lines in the 270 deg direction diverge and the average estimate was 266.4 deg. We find that saccade direction estimates derived from the LFP generally hovered close to the actual direction; for the F1 data set, average estimates of the saccade direction (dashed lines) had errors ranging from 0.8 deg to 18.5 deg. It is important to note the difference in meaning of the AUCs in this figure vs. Figures 7, 8. For instance, the AUCs in Figures 7, 8 are high in the 30-degree saccade direction; this indicates that with saccades in that direction the SVM can reliably (high AUC) classify LFPs as coming from a saccade or fixation period of time. In Figure 10 the AUCs in the 30-degree direction are lower using the one-direction vs. all-other directions classifier. This difference arose because the LFPs in the 30- and 60-deg directions were similar and some of the 30-deg saccades were mistakenly classified as 60-deg saccades even though saccade LFPs for both directions were quite different from fixation LFPs.

Based on the data in Figure 10, it appears that area V1 receives signals conveying information about saccade metrics; saccade direction can be inferred accurately on many channels and for multiple directions. The errors in saccade direction estimates are summarized in Table 1. Consistent with the data plotted in Figure 10, average direction estimates were close to the actual direction and there was considerable variance in the direction estimates across saccade directions; performance on some channels was spot-on in certain directions but other directions gave significantly higher errors.

**Table 1:** Saccade direction error-estimates in the F1, F2, and S data sets.

	Average error (deg)	RMS error (deg)	Error Std Dev (deg)	Min/Max error (deg)
F1	8.0	8.0	10.6	0.89/18.5
F2	8.3	8.3	10.6	0.43/18.1
S	24.1	24.2	28.9	2.9/48.7

## Discussion

Findings in this study build upon previous reports that brain activity changes around the time of saccades. Prior research has investigated the extent to which activity in visual areas changes based on “where the eyes are” and “what the eyes are doing.” Neurons in multiple visual areas carry information about eye position (Sakata et al., 1980; Andersen and Mountcastle, 1983; Galletti and Battaglini, 1989; Merriam et al., 2013; Morris et al., 2013). More closely related to the present study are experiments investigating temporal changes in brain activity associated with saccades. Inflections in occipital EEGs (lambda waves) are observed when saccades are made across complex images (Evans, 1953; Roth and Green, 1953; Yagi, 1979; Thickbroom et al., 1991; Brigo, 2011). There are several similarities between our data and lambda waves: they span similar peri-saccadic time periods, they occur without visual stimulation, and they are sensitive to saccade direction (Skrandies and Laschke, 1997).

Specifically concerning areas V1 and V2, multiple studies report neural activity related to our findings. For example, there is pre-saccadic response enhancement that appears to be associated with oculomotor planning and/or attention (Wurtz and Mohler, 1976; Boch, 1986; Super et al., 2004). Duffy and Burchfiel (1975) reported post-saccadic inhibition of V1 single unit activity that was directional and present in complete darkness (indicating an extra-retinal signal). Purpura et al. (2003) recorded V2 eye-movement related potentials that began at or slightly after saccade onset and continued for over 100 ms into the following fixation; direction sensitivity was occasionally observed. Rajkai et al. (2008) recorded multiunit V1 activity in complete darkness and found neural suppression during saccades followed by increased activity after fixations begin. This response pattern is similar to the LFPs we observed. Saccade-related signals have also been observed in human V1 using fMRI and these signals are present even with saccades in the dark (Bodis-Wollner et al., 1997; Sylvester et al., 2005; Rajkai et al., 2008).

In summary, previous research establishes that there are peri-saccadic changes in brain activity across multiple brain areas and that these signals do not rely on visual input. The study described here is novel in its quantification of the information available in the peri-saccadic signals. It should be noted that our performance estimates could almost certainly be improved upon if modifications were made, such as an SVM incorporating nonlinear separating hyperplanes or nonlinear combinations of channels or using a different machine learning approach.

We found that an SVM can reliably classify portions of the LFP as saccade or fixation; performance was good when saccade-related LFPs were classified relative to LFPs from pre-saccadic or post-saccadic fixations (i.e., both saccade-to-fixation and fixation-to-saccade transitions can be reliably detected). The LFP also provides information sufficient to accurately determine when a new fixation begins: under optimal conditions, single channels make it possible to estimate fixation onset with an error less than a few milliseconds. The temporal location of the training window played a key role in the precision of the fixation onset estimates. The best estimates of fixation onset time were obtained using LFPs in windows ranging from about τ = −20 ms to the latest points analyzed at τ = 49 ms. Classification performance varied with saccade direction and, interestingly, was greatest when saccades moved the eyes in the direction toward RFs. This finding may have implications for interactions with the superior colliculus, the relationship between saccades and attention, and forward vs. convergent remapping (Neupane et al., 2016).

Though there were variations within and across channels, fixations and saccades could be classified regardless of saccade direction. Classification performance declined with shorter analysis windows but AUCs in the range of 0.8–1.0 were obtained even with optimal 10 ms analysis windows. The optimal 10 ms windows had center times ranging from shortly before and up to about 30 ms after fixation onset. The high AUCs, obtained with LFPs in short temporal windows near the ends of saccades, suggest that the brain could conceivably monitor the LFP for a change from the saccade pattern and determine within about 10 ms that a new fixation has started. It is noteworthy that classification performance well above chance is obtained even with 10 ms of LFP activity that is purely presaccadic. Hence it appears that eye movement information reaches V1 before the eyes start moving; information then increases during and after the saccade.

SVM classifiers were also made to assess the ability to use the LFP to infer the direction of a saccade. As illustrated in Figure 10, the direction estimates were generally clustered around the correct saccade direction; LFPs recorded on some of the electrodes carried remarkably accurate information about saccade direction. To assess aspects of the LFPs that might be responsible for the classification performance, we considered several factors. The peri-saccadic LFP in Figure 1 has a distinctive shape but it also has a higher mean amplitude than fixation intervals. This raises the question whether classification performance is based on the shape or simply the amplitude of the LFP. Figure 2 showed examples of comparisons we made of classification performance with a 100-dimensional SVM and a simple classifier based on mean LFP amplitude. Though there were situations in which the mean classifier approached the performance of the SVM, in general the AUC measures based on mean-amplitude classification were far below AUCs obtained with the 100-dimensional SVM. This demonstrates the importance of the temporal shape of the LFP for classification. That said, there does not appear to be a single peri-saccadic LFP shape that is useful for saccade classification; as shown in Figure 6, the LFP varied considerably with saccade direction and a range of LFP shapes were associated with high AUC values.

### Corollary Discharge

The LFP modulation and associated saccade information we have observed appear to be extraretinal rather than a result of visual stimulation. As mentioned above, similar LFPs in area V1 were reported by Rajkai et al. (2008) in complete darkness, demonstrating a general dissociation from visual stimulation. In our experiments, we chose to have animals make saccades to small fixation points so that repeated saccades could be made and signals averaged with controlled saccade metrics. Multiple factors suggest that the results in our study were not based on visual stimulation by the fixation points or stimuli beyond the visual display. The RFs we studied were generally less than 1 deg in diameter and 3.6–4.5 deg eccentric, approximately along a 45 deg diagonal, down and to the left of fixation. The saccade directions that took the RFs closest to the fixation point were in the 30 and 60 deg directions but, even in these directions, the spots never entered the classical RFs measured with spiking activity. There is disagreement about the lateral spread of LFPs in V1 (e.g., Berens et al., 2008; Katzner et al., 2009) but there is compelling evidence that V1 RFs measured with LFPs are similar in size to RFs measured with spiking activity (Xing et al., 2009). Two additional observations make it even less likely that visual stimulation during the saccade produced the perisaccadic LFPs we recorded. First, the 0.25 deg spots used as fixation points were generally poor stimuli for the neurons under study, usually evoking no action potentials even when the eyes were static. Second, at the closest approach of the RF to the fixation spot, the eye speed was 300–350 deg/s, well outside the pass band of V1 neurons (e.g., preferred speeds in Priebe et al., 2006 were 0.3–43 deg/s with tuning bandwidths around 1.5 octaves). Two factors suggest that stimuli beyond the visual display did not affect the results. First, the RFs were small (<1 deg diameter) and there was always a significant distance between the RFs and the edge of the display which was 33 deg wide by 26 deg tall. On the final fixation, the RFs were approximately 10 deg from the lower edge of the display and 13 deg from the left side of the display. At the 12 “pre-saccadic” fixations, the RF distance ranged 3–17 deg from the bottom of the display and 7–21 deg from the left side of the display. Moreover, in early experiments (Ruiz et al., 2010), a large uniformly illuminated foam core panel (78 deg wide by 65 deg high) surrounded the visual display. Subsequent experiments were conducted without the large surround panel as it was found to have no influence on the data.

As the LFP modulations we recorded were not visually driven, it appears they reflect an eye-movement corollary discharge (CD) that projects to area V1. We find that saccade occurrence, timing, and direction can be inferred well above chance from the LFPs, even before a saccade begins. The early timing rules out visual and proprioceptive inputs, at least as the sole source of the signals. Further evidence consistent with a CD comes from the finding that peri-saccadic signals are similar in light and darkness, hence, not dependent on visual input (Bodis-Wollner et al., 1997; Skrandies and Laschke, 1997; Sylvester et al., 2005; Rajkai et al., 2008). Our data show that the high classification performance was obtained with LFP signals in a fairly broad peri-saccadic time span. It is not clear whether such a sustained signal is appropriate for CD, but it may be relevant that RF remapping in individual frontal eye field neurons, thought to rely on CD, starts over an even broader time period (Kusunoki and Goldberg, 2003; Sommer and Wurtz, 2006). Alternatively, later inputs reaching V1 might come from proprioceptive signals that are delayed relative to changes in eye position (Wang et al., 2007; Xu et al., 2011). However, there is no evidence that proprioceptive input carries the precise information we have quantified or that the V1 LFP signals reflect two types of input.

The presence of a CD signal in V1 raises the question of its source. Sommer and Wurtz (2002) traced a CD pathway from the superior colliculus to the mediodorsal nucleus of the thalamus and on to the frontal eye fields. Mediodorsal nucleus projections are extensive in the frontal lobe but they do not include early visual cortical areas (Giguere and Goldman-Rakic, 1988). CD signals may reach V1 via topographic projections from the superior colliculus to the pulvinar nucleus of the thalamus (Campos-Ortega and Hayhow, 1972; Benevento and Rezak, 1976; Rezak and Benevento, 1979; Adams et al., 2000). Recordings in both the superior colliculus (Richmond and Wurtz, 1980) and the pulvinar (Robinson and Petersen, 1985) are consistent with CD. Further evidence for interactions between the pulvinar and visual cortex come from studies of visual attention (Zhou et al., 2016) and the surprisingly powerful suppressive effect that inactivation of the lateral pulvinar has on V1 activity (Purushothaman et al., 2012).

### Significance for Vision

There are several important visual functions that might make use of temporal and directional information about saccades that reaches V1. An example that has received considerable attention is visual stability. Going back centuries, it has been speculated that the brain must know what the eyes are doing to avoid the interpretation that the world moves with each saccade (Helmholtz, 1866/1911; Grüsser, 1995; Cavanaugh et al., 2016). Conceivably, the V1 signals we studied could be used in this compensation process (in V1 or in a later area)—the LFP in V1 shows rapid and reliable changes that could be used to infer the timing of saccade onset and offset and the direction of the saccade that moved the eyes. This information is most reliably represented in larger post-saccadic temporal windows, but it is noteworthy that significant information about saccade timing and direction is observed even in brief windows before and during saccades. A component of visual stability may be the loss of sensitivity to visual input that occurs during saccades—i.e., saccadic suppression (Matin, 1974; Ross et al., 2001). Signals in V1 could be used to suppress visual input either as the saccade occurs or through backward masking with a post-saccadic signal.

The continuous alternation between saccades and fixations, characteristic of natural vision, influences processing in several ways and a signal to identify fixation onset may facilitate these processes. For example, Rajkai et al. (2008) saw phase resetting at the start of new fixations and hypothesized that a “fixation amplifier” may enhance visual processing. Early fixation spikes are also phase-locked to LFP modulations suggesting that the eye movement and associated LFP affect spike coding (Maldonado et al., 2008; Ito et al., 2011). As much of visual recognition appears to be based on a rapid feedforward sweep of neural activity (Keysers et al., 2001; VanRullen and Thorpe, 2002), it may be critical that new fixations can be rapidly detected. In macaque V1, saccades produce biphasic modulation of the spiking responses to visual stimuli (McFarland et al., 2015). In human psychophysics experiments, Paradiso et al. (2012) showed that a saccadic eye movement decreases the integration of information from one fixation to the next and De Pisapia et al. (2010) found that after the start of a new fixation there are alternating periods of integration and segregation. The common thread in many of these studies is that visual analysis synced to fixation onset may enhance visual processing and perception. The present results demonstrate that V1 has such information and with high enough temporal precision, to be a significant factor in visual processing.

## Data Availability

The raw data supporting the conclusions of this manuscript will be made available by the authors, without undue reservation, to any qualified researcher.

## Author Contributions

The experiments and analyses in this study were conceptualized by all the authors. Physiological data was recorded by OR and JN; data analysis was performed by SA-C.

## Conflict of Interest Statement

The authors declare that the research was conducted in the absence of any commercial or financial relationships that could be construed as a potential conflict of interest.
